# New Dental Society

**Published:** 1870-09

**Authors:** 


### NEW DENTAL SOCIETY.

A new Dental society was organized at Maysville, Ky., on Tuesday, Sept. 6, 1870, including members of the profession in North-eastern Kentucky and Southern Ohio.

Dr. H. A. Beamer, of Carlisle, Ky., was chosen temporary chairman, and after preliminaries were settled the venerable Dr. McCullum, of Augusta, was elected President; Dr. M. Bratt, of Maysville, Vice President, and Dr. W. Vanantwerp of Mt. Sterling, Treasurer.

A committee of three was appointed to read essays, and a committee of three to frame a Constitution and Code of Ethics. A Preamble to the Constitution, setting forth the objects of the Association and naming it the Ohio Valley Dental Association, was accepted. It was also agreed that the Association should meet semi-annually, or in the months of May and October.

The new Association then adjourned to meet at Augusta, in May, 1871.

